# Seroepidemiology of SARS-CoV-2 infections in an urban population-based cohort in León, Nicaragua

**DOI:** 10.1017/S0950268821002144

**Published:** 2021-10-20

**Authors:** Fredman González, Nadja A. Vielot, Michael Sciaudone, Christian Toval-Ruíz, Lakshmanane Premkumar, Lester Gutierrez, Edwing Centeno Cuadra, Nancy Munguia, Patricia Blandón, Aravinda M. de Silva, Rebecca Rubinstein, Natalie Bowman, Sylvia Becker-Dreps, Filemon Bucardo

**Affiliations:** 1 Center for Infectious Diseases Research, National Autonomous University of Nicaragua – León, León, Nicaragua; 2 Department of Family Medicine, University of North Carolina at Chapel Hill, Chapel Hill, North Carolina, USA; 3 Division of Infectious Diseases, University of North Carolina at Chapel Hill, Chapel Hill, North Carolina, USA; 4 Department of Microbiology and Immunology, University of North Carolina at Chapel Hill, Chapel Hill, North Carolina, USA; 5 School of Medicine, University of North Carolina at Chapel Hill, Chapel Hill, North Carolina, USA

**Keywords:** COVID-19, epidemiology, Nicaragua, SARS-CoV-2, seroprevalence

## Abstract

In a Nicaraguan population-based cohort, SARS-CoV-2 seroprevalence reached 28% in the first 6 months of the country's epidemic and reached 35% 6 months later. Immune waning was uncommon. Individuals with a seropositive household member were over three times as likely to be seropositive themselves, suggesting the importance of household transmission.

## Introduction

SARS-CoV-2 transmission continues globally [1]. The pandemic is now entering a prolonged phase, potentially causing multiple waves of infection in populations that have not attained community immunity. Information on SARS-CoV-2 seroprevalence in different populations is urgently needed to understand the magnitude of SARS-CoV-2 spread in previous waves, predict future waves and measure the risk of re-infection in previously exposed persons, particularly in light of the emergence of highly-transmissible SARS-CoV-2 variants [2, 3]. Furthermore, few studies have examined SARS-CoV-2 seroprevalence in population-based samples with substantial representation from pre-school and school-aged children, or in individuals with factors which place them at higher risk for poor outcomes [4–6].

Prior studies have measured SARS-CoV-2 seroprevalence in hotspots that were heavily impacted by infections in the first wave of the pandemic (December 2019–May 2020). In Wuhan, China, where the pandemic originated, seroprevalence was 4% in May 2020; in Lombardy, Italy, seroprevalence among blood donors reached 23% by April 2020 [7, 8]. This wide range of estimates suggests that geographical, social and economic differences, as well as the implementation of containment measures, can greatly affect exposure to and infection with SARS-CoV-2.

Even less is known in low- and middle-income countries (LMICs), which generally have limited resources to perform molecular detection of SARS-CoV-2 in real-time, complicating estimates of incident infection and individual disease risk. While global SARS-CoV-2 case detection has improved since the start of the epidemic, some countries continue to face barriers to routine surveillance and case reporting. Furthermore, LMICs have fewer resources to support remote work and schooling, hygiene measures and vaccines, and households are often multi-generational. This study aimed to estimate the seroprevalence of SARS-CoV-2 in a population-based sample in León, Nicaragua at 6 and 12 months since SARS-CoV-2 infections were first reported in March 2020. In the first year of the pandemic, infection data from Nicaragua were not consistently available in global databases, leaving a knowledge gap with respect to how to prevent outbreaks in this context [9, 10]. These data on the prevalence of past infections can be used to guide public health recommendations and inform the need for the continuation of SARS-CoV-2 prevention measures.

## Methods

### Study design and population

The Sapovirus-Associated Gastro-Enteritis (SAGE) study is a population-based birth cohort study in León, Nicaragua, described previously [11]. This cohort provided a platform to access a sample of all ages from public health facility records, capturing a large and representative sample of this large urban area to understand the seroprevalence of SARS-CoV-2 in a Nicaraguan context, and to examine differences in participant characteristics by evidence of prior infection. The study population included high-income families in the city centre and low-income families in peri-urban neighbourhoods, creating a scientifically-informative gradient to evaluate socioeconomic and environmental risk factors for SARS-CoV-2 infection. Starting in July 2020, we contacted the household members of 350 cohort children (both adults and children) and offered participation in this study. We also offered participation in the study for cohort children, including those who had reached 36 months of age. Any interested household members were eligible to enrol, and not all household members were required to enrol. All adult participants provided informed consent, and parental consent plus child's assent was required for children ages 7–17 years. The study was approved by the Institutional Review Boards of the National Autonomous University of Nicaragua, León (UNAN-León, acta No. 170, 2020) and the University of North Carolina at Chapel Hill (Study #: 20-2126).

In September and October 2020, we collected baseline demographic and health history data and collected serum from all participants for baseline SARS-CoV-2 serology. We requested a second serum sample from participants in February and March 2021. The current report summarises the seroprevalence of SARS-CoV-2 at 6 and 12 months since the virus was first detected in Nicaragua, stratifying by co-morbidities and sociodemographic factors.

### Specimen collection and laboratory methods

SARS-CoV-2 infection induces the production of antibodies (Ab) against the spike protein and nucleocapsid protein, with most patients seroconverting within 2 weeks of symptom onset [12]. We used a previously-validated in-house enzyme-linked immunosorbent (ELISA) assay to measure total antibodies (IgG, IgA and IgM) to the receptor-binding domain (RBD) of the SARS-CoV-2 spike protein. This assay was shown to be 95.2% sensitive and 96.7% specific for detecting antibodies among individuals experiencing symptomatic infection at 9 days since symptoms onset [13]. The spike RBD-based assay does not cross-react with common human endemic coronaviruses, and the magnitude of RDB antibody levels correlates with neutralising antibody titres, currently the best correlate of protection against infection. In brief, ELISA plates (Grenier Bio One: Monroe, North Carolina, USA) were coated with 4 μg/ml of the RBD antigen. Heat-inactivated serum diluted at 1:40 was subsequently added and alkaline phosphate conjugated secondary goat anti-human Abs (anti-IgG (MilliporeSigma: Burlington, Massachussetts, USA), anti-IgA (Abcam: Cambridge, United Kingdom) and anti-IgM (Sigma)) were added at 1:2500 dilution for detection. The immunologic reaction was developed with para-nitrophenyl phosphate substrate (Sigma). The optical density (OD) was measured after 15 min at 405 nm. We previously tested a large panel of known SARS-2 antibody-negative and positive samples and established the OD cut-off that separates negative from positive samples [12]. At a single serum dilution of 1:40, OD > 0.3 was a sensitive and specific indicator of SARS-2 RBD antibodies. A serum was considered seropositive if the absorbance of positive/negative ratio was ≥2.57, to ensure 99.5% specificity per CDC guidelines [13, 14].

### Statistical analysis

We analysed cross-sectional data using frequencies and percentages to characterise SARS-CoV-2 seroprevalence at each time point, stratified by sex, age group, smoking status, presence of comorbidities and socioeconomic characteristics of the household. The SARS-CoV-2 seroprevalence at each time point was estimated directly based on the ELISA results as the ratio of the number of seropositive samples to the number of samples tested. Bernoulli 95% confidence intervals were estimated using the Clopper-Pearson exact method. Seroprevalence was also adjusted to account for imperfect test performance, assuming 95.2% sensitivity and 96.7% specificity, using the Bayesian method described by Bendavid *et al* [15]. We implemented generalised estimating equations to estimate prevalence ratios (PRs) comparing seropositivity proportion by select individual and household characteristics, accounting for clustering of individuals within households. Data were analysed using SAS version 9.4 (SAS Institute, Cary, North Carolina, USA) and R (R Foundation for Statistical Computing, Vienna, Australia). Epidemiologic and lab data from the SAGE are available by request to the corresponding author (FB) with permission from the principal investigators.

## Results

In July 2020, we began contacting 1846 individuals from 297 households actively enrolled in the SAGE study to assess their interest in the current study. Between September and October 2020, 1381individuals from 279 households agreed to participate. Of 1243 individuals who provided serum samples at both time points, 347 (27.9%) were seropositive for SARS-CoV-2 at the first time point, and 437 (35.2%) were seropositive at the second time point based on the results of the ELISA assays. Adjusting for test performance, the seroprevalence was 26.8% (95% CI 23.3–30.1%) at the first time point and 34.7% (95% CI 31.2–38.2%) at the second time point. At enrolment, at least one household member was seropositive for SARS-CoV-2 in 51.9% of households (*n* = 153), with a median 2 seropositive household members (interquartile range: 1–4, max: 12). At the 6-month follow-up, the proportion of households with at least one seropositive member increased to 60.3% (*n* = 178).

At enrolment, over 40% of seropositive individuals were younger than 15 years (Table 1). By the 6-month follow-up visit, seroprevalence was slightly more balanced among the age groups, ranging from 33% to 36% among those under 70 years of age (Table 1). The presence of comorbidities was associated with a lower prevalence of seropositivity as baseline, and was not associated at 6-month follow-up; however, these associations are imprecise due to small numbers and should be interpreted with caution. Seropositivity was not associated with physical distancing or the use of facial coverings nor with economic status of the household. Seropositivity was strongly associated with living with another seropositive household member, conferring a nearly fourfold prevalence at enrolment (PR 3.94, 95% CI 2.80–5.55) and a greater than threefold prevalence at 6 months (PR 3.26, 95% CI 2.32–4.60) (Table 2).

**Table 1.** SARS-CoV-2 seropositivity of individuals in León, Nicaragua in the first year of the epidemic, by selected characteristics (*n* = 1243)

	Seroprevalence (95% confidence interval)			
	September–October 2020		February–March 2021	
Individual and household characteristics	Crude	Adjusted^a^	Crude	Adjusted^a^
Sex (*n* = 1240)				
Male	30.0 (26.0, 34.2)	29.2 (24.1, 33.9)	36.2 (32.0, 40.6)	44.3 (39.9, 48.7)
Female	26.5 (23.3, 29.8)	25.2 (21.3, 29.4)	34.3 (30.9, 37.9)	33.8 (29.7, 38.4)
Age group at enrolment, years (*n* = 1216)				
0–4	29.4 (24.5, 34.7)	28.5 (22.5, 34.3)	33.8 (28.6, 39.2)	33.3 (27.4, 39.0)
5–14	29.0 (23.2, 35.3)	29.6 (22.3, 37.0)	35.5 (29.3, 42.1)	35.5 (28.8, 42.3)
15–29	27.4 (22.8, 32.4)	26.3 (21.2, 31.7)	36.0 (31.0, 41.3)	35.7 (29.8, 41.7)
30–49	23.0 (17.4, 29.5)	21.4 (14.6, 28.1)	33.0 (26.5, 40.0)	32.4 (25.1, 39.9)
50+	27.8 (19.9, 37.0)	27.0 (18.6, 36.0)	37.4 (28.6, 46.9)	37.3 (27.7, 46.5)
Smoking status (*n* = 618)^b^				
Never smoker	26.3 (22.6, 30.3)	19.1 (10.5, 29.1)	35.4 (31.3, 39.7)	29.6 (18.9, 40.2)
Ever smoker	20.0 (12.3, 29.8)	25.0 (20.3, 29.7)	30.0 (20.8, 40.6)	35.0 (29.8, 39.9)
Presence of comorbidities (*n* = 1208)^c^				
No	27.7 (25.0, 30.4)	26.5 (22.6, 30.0)	34.5 (31.7, 37.4)	34.0 (30.1, 37.9)
Yes	25.0 (17.4, 33.9)	24.2 (15.8, 32.8)	35.3 (26.7, 44.8)	35.2 (25.8, 46.0)
Diabetes (*n* = 116)				
No	25.0 (16.6, 35.1)	24.0 (15.1, 34.5)	34.8 (25.2, 45.4)	34.8 (24.5, 46.1)
Yes	25.0 (9.8, 46.7)	26.0 (10.8, 44.8)	37.5 (18.8, 59.4)	38.3 (19.4, 58.3)
Hypertension (*n* = 116)				
No	21.4 (11.6, 34.4)	20.4 (9.0, 32.9)	28.6 (17.3, 42.2)	28.4 (16.5, 42.0)
Yes	28.3 (17.5, 41.4)	28.0 (15.7, 32.2)	41.7 (29.1, 55.1)	42.7 (29.3, 55.6)
Malignancy (*n* = 116)				
No	24.5 (17.1, 33.8)	23.6 (15.0, 32.0)	35.4 (26.6, 45.0)	35.2 (25.3, 45.5)
Yes	33.3 (0.84, 90.6)	39.3 (4.5, 83.3)	33.3 (0.84, 90.6)	39.3 (4.5, 83.3)
Autoimmune disease (*n* = 116)				
No	25.7 (17.9, 34.7)	24.7 (15.8, 33.5)	35.4 (26.6, 45.0)	35.2 (25.3, 45.5)
Yes	0	21.1 (0.6, 62.0)	33.3 (0.84, 90.6)	39.3 (4.5, 83.3)
Physical distancing and use of facial coverings (*n* = 1215)^d^				
Strict	28.2 (25.5, 31.0)	27.1 (23.7, 30.5)	35.2 (32.4, 38.1)	38.4 (30.9, 38.7)
Moderate	34.8 (21.4, 50.3)	35.1 (21.3, 49.6)	43.5 (28.9, 58.9)	44.5 (29.3, 59.2)
None	25.0 (15.8, 36.3)	24.3 (14.2, 35.5)	32.9 (22.5, 44.6)	32.5 (21.5, 44.8)
Crowding index (*n* = 1118)				
Fewer than 2.7 people per bedroom	28.1 (24.9, 31.5)	26.9 (23.0, 31.0)	37.0 (33.5, 40.6)	36.7 (32.4, 41.2)
2.7 or more people per bedroom	26.5 (22.2, 31.2)	25.2 (19.9, 30.6)	30.1 (25.6, 35.0)	29.5 (24.0, 35.0)
Poverty index (*n* = 1118)^e^				
Not poor	27.0 (23.2, 31.0)	25.8 (21.4, 30.9)	35.2 (31.0, 39.5)	34.9 (29.7, 40.0)
Poor	29.0 (25.0, 33.2)	28.0 (23.4, 32.8)	35.6 (31.4, 40.0)	35.4 (30.1, 40.8)
Extremely poor	24.1 (16.5, 33.1)	22.8 (14.4, 32.3)	27.7 (19.6, 36.9)	26.8 (18.2, 35.8)
Household member is seropositive				
No	12.3 (9.6, 15.4)	9.7 (5.6, 13.6)	15.5 (12.1, 19.6)	13.3 (8.1, 18.1)
Yes	39.1 (35.6, 42.8)	39.1 (34.5, 43.7)	44.0 (40.6, 47.4)	44.3 (39.9, 48.7)

a Adjusted for imperfect test performance (sensitivity = 95.2%; specificity = 96.7%).

b Smoking status assessed among participants older than 13 years of age.

c Diagnosed by medical professionals as reported by participant, including diabetes, hypertension, obesity, malignancy and autoimmune disease.

d Strict = never left the house since first cases reported or occasionally leaves house and always uses a mask; moderate = occasionally leaves the house and sometimes uses a mask; none = occasionally leaves the house and never uses a mask or no distancing or quarantine behaviour.

e Poverty index calculated based on the presence of basic needs, including household sanitation, education, economic dependency and household crowding.

**Table 2.** Characteristics associated with SARS-CoV-2 seropositivity of individuals in León, Nicaragua in the first year of the epidemic (*n* = 1243)

	Prevalence ratio (95% confidence interval)^a^	
Individual and household characteristics	September–October 2020	February–March 2021
Sex (*n* = 1240)		
Male	1.0 (ref)	1.0 (ref)
Female	0.92 (0.76, 1.12)	0.96 (0.81, 1.12)
Age group at enrolment, years (*n* = 1216)		
0–4	0.96 (0.90, 1.03)	1.00 (0.95, 1.06)
5–14		
15–29		
30–49		
50–69		
70 +		
Smoking status (*n* = 618)^b^		
Never smoker	1.0 (ref)	1.0 (ref)
Ever smoker	0.79 (0.54, 1.16)	0.87 (0.62, 1.22)
Presence of comorbidities (*n* = 1208)^c^		
No	1.0 (ref)	1.0 (ref)
Yes	0.89 (0.63, 1.25)	1.00 (0.74, 1.35)
Physical distancing and use of facial coverings (*n* = 1215)^d^		
Strict	1.01 (0.86, 1.20)	1.04 (0.89, 1.21)
Moderate		
None		
Crowding index (*n* = 1118)		
Fewer than 2.7 people per bedroom	1.0 (ref)	1.0 (ref)
2.7 or more people per bedroom	0.96 (0.72, 1.27)	0.82 (0.64, 1.04)
Poverty index (*n* = 1118)^e^		
Not poor	0.99 (0.82, 1.20)	0.94 (0.79, 1.11)
Poor		
Extremely poor		
Household member is seropositive		
No	1.0 (ref)	1.0 (ref)
Yes	3.94 (2.80, 5.55)	3.26 (2.32, 4.60)

a Based on crude seroprevalence estimates.

b Smoking status assessed among participants older than 13 years of age.

c Diagnosed by medical professionals as reported by participant, including diabetes, hypertension, obesity, malignancy and autoimmune disease.

d Strict = never left the house since first cases reported or occasionally leaves the house and always uses a mask; moderate = occasionally leaves the house and sometimes uses a mask; none = occasionally leaves the house and never uses a mask or no distancing or quarantine behaviour.

e Poverty index calculated based on the presence of basic needs, including household sanitation, education, economic dependency and household crowding.

Among the 347 individuals who were SARS-CoV-2 seropositive at enrolment, 327 (adjusted 97.9%, 95% CI 94.7–99.9) remained seropositive at 6-month follow-up. In the remaining 20 individuals, antibody levels to spike RBD decreased below the detection level at 6 months. Among the 896 individuals who were SARS-CoV-2 seronegative at enrolment, 110 individuals (9.7%, 95% CI 6.3–13.1) seroconverted at 6 months.

## Discussion

Our results show a 28% seroprevalence of SARS-CoV-2 antibodies in a population-based cohort in an urban area of Nicaragua 6 months after the first case in the country was reported. Seroprevalence at the end of the first year of the epidemic was 35%, suggesting that the actual number of SARS-CoV-2 infections in Nicaragua was higher than official reports. This might be explained by asymptomatic infections that did not seek medical attention, or by limited testing of patients who received medical attention. Meta-analyses have estimated that more than one-third of SARS-CoV-2 infections are asymptomatic [16]. The spike RBD Abs remained detectable over 6 months, which is consistent with other studies that showed anti-SAR cS-CoV-2 spike protein IgG remains detectable in most cases for at least 6 months after symptomatic infection [17].

Our seroprevalence results are similar to those reported from SARS-CoV-2 hot spots in urban areas of high-income countries, such as Massachusetts and Northern Italy, which reached 24–28% within 2 months of identification of the first COVID-19 cases [7, 18]. While the Massachusetts study was not population-based, these findings suggest that urban areas are vulnerable to high SARS-CoV-2 transmission rates. In these studies, the seroprevalence of SARS-CoV-2 antibodies was higher than expected based on the reported incidence of symptomatic SARS-CoV-2 infections in the area. It is possible we slightly underestimated the true number of SARS-CoV-2 cases because of the high positive/negative cut-off used to ensure high assay specificity. A study of outpatients seeking care for acute respiratory disease during the first epidemic wave of SARS-CoV-2 in León showed that 38% had an acute mild COVID-19 infection [19]. A recent cross-sectional study of Nicaraguan health care workers (HCWs) conducted over 1 month found that 30% of participants had active SARS-CoV-2 infection based on the amplification of the SARS-CoV-2 RNA in saliva samples. When accounting for resolved infections in which virus would not be detected, it is likely that SARS-CoV-2 seroprevalence among HCWs is even higher than the infection prevalence, suggesting that seroprevalence among Nicaraguan HCWs is likely higher than in the general population [20]. In the same HCW study, about half of SARS-CoV-2 infected HCWs were asymptomatic, and, similar to our results, men were more likely to have been infected in the initial stages of the epidemic [17]. The use of personal protective equipment is critical among HCWs, both to prevent contracting SARS-CoV-2 from infected patients and to prevent transmitting SARS-CoV-2 to patients, particularly when the HCW is asymptomatic.

At enrolment, we identified high seroprevalence among younger age groups, particularly in children between 5 and 14 years of age (46%). Age-based differences in seropositivity were observed in Brazil and Italy [7, 21]; however, they were more pronounced in our population. High seropositivity in children might be due to continued operation of public schools throughout the epidemic, which might have promoted SARS-CoV-2 spread in classrooms, cafeterias and other group spaces. As children are more likely to experience mild symptoms than adults and are thus less likely to self-isolate [22], school-aged children may contribute to active transmission to other children, and subsequently to adults and others within the household. By the 6-month follow-up visit, seroprevalence was more balanced among the age groups, suggesting that child-to-adult transmission occurred primarily in the early stages of the epidemic. We also found that most individuals reported practicing physical distancing or wearing facial coverings outside of the home, though this did not have a significant impact on the seroprevalence. Transmission within the home may have been more important [23]. The median household size in our sample was seven members, with a maximum of 26 members. Indeed, SARS-CoV-2 seropositivity was up to four times as prevalent in individuals with a seropositive household member as compared to individuals who did not have a seropositive household member [24–26]. Prevention of household transmission, particularly in low-income settings that experience household crowding and other social inequities that increase infection risk, is a critical component of COVID-19 management worldwide [24].

This study provides insights on disease burden in Central America, where disease surveillance may be limited and may have important public health implications due to the high rates of migration within and outside of the Central America [27]. Limitations of this study include inability to enrol and collect samples from all household members, which may underestimate the number of seropositive individuals within households. In addition, limited resources precluded real-time diagnosis of SARS-CoV-2 infections by RT-qPCR, which could have been used to validate and provide context to the seroprevalence results. Finally, the high performance of the immunological assay as reported by Premkumar *et al*. may not reflect the assay's performance in the current study, as the assay was validated in symptomatic individuals with confirmed SARS-CoV-2 infection. The assay is less sensitive in the first 8 days since symptoms onset, and it is unclear how the assay performs in asymptomatic individuals [12]. However, the primary strength of this study is the population-based sampling framework and the ability to identify infection trends within households, including a variety of age groups. In the future, this population could be followed to understand the risk of recurrent infection, and repeated seroprevalence measurements could provide further information on transmission dynamics. Finally, the correlates of protection against SARS-CoV-2 infection could be established by capturing incident infections with SARS-CoV-2 within the cohort.

In conclusion, we found a high SARS-CoV-2 seroprevalence in this Nicaraguan population and confirmed that reported SARS-CoV-2 case counts underestimated the true number of infections. We also show that this population has not yet attained the theoretical community immunity threshold. Thus, continued prevention and containment measures are necessary. In March 2021, the Nicaraguan Ministry of Health began vaccination campaigns targeted at adults 65 years and older and those with chronic conditions, providing immunity to the most vulnerable citizens [28]. While data on the percentage of the population who is vaccinated are not publicly available, our data suggest that antibody responses tend to persist for at least 6 months, and that community immunity is theoretically attainable through a combination of natural infection and vaccination.

## Data Availability

Epidemiologic and lab data from the SAGE are available by request to the corresponding author (FB) with permission from the principal investigators
